# The Relationship Between Perfectionism and Sports Ethics Among Young Athletes Based on Achievement Goal Theory

**DOI:** 10.3389/fpsyg.2022.771332

**Published:** 2022-03-10

**Authors:** Kaihong Sun, Tai Ji

**Affiliations:** ^1^Department of Physical Education, Changzhou Vocational Institute of Textile and Garment, Changzhou, China; ^2^Department of Physical Education, Yangzhou Polytechnic College, Yangzhou, China; ^3^Department of Physical Education, Shanghai Jiao Tong University, Shanghai, China

**Keywords:** perfectionism, ethic, athletes, achievement goal, SEM

## Abstract

Exercise plays an important role in the process of socialization among young people and children by providing a context in which children can be exposed to the existing rules and values of society. However, the increasing news of unethical behaviors reported in competitive scenarios led the public to suspect the view “sports shape great characters.” To investigate the issue and explore potential influencing factors, the study examined the relationship among athletes’ perfectionism, achievement goals, and sports ethics based on the achievement goal theory. A total of 243 young athletes were recruited. The multidimensional perfectionism, achievement goals, and sports ethics were measured by a battery of questionnaires. A direct effect model that did not include mediation variables (achievement goals) and a mediation effect model that included mediation variables were analyzed by structural equation modeling (SEM). Results indicated that perfectionistic strivings may positively predict sports ethics, whereas perfectionistic concerns may negatively predict sports ethics. Mastery approach and mastery avoidance may positively predict sports ethics, whereas the performance approach and performance avoidance may negatively predict sports ethics. Achievement goals may partially mediate the relationship between perfectionism and sports ethics. Perfectionistic strivings may negatively predict sports ethics through performance approach and positively predict sports ethics through mastery approach. Perfectionistic concerns may negatively predict sports ethics through performance avoidance and positively predict sports ethics through mastery avoidance. To prevent athletes from using unethical behaviors, strategies should be developed to reduce perfectionistic concerns and increase their perfectionistic strivings.

## Introduction

Exercise plays an important role in the process of socialization since it provides a context in which individuals can be exposed to the existing rules and values of reality society (Evans and Roberts, 1987). In those contexts, participation in sports is considered as a vehicle of learning and cultivating how to cooperate, negotiate, and resolve moral conflicts and how to be courageous, fair, loyal, and persistent (Shields and Bredemeier, 1995). However, the increasing news of unethical behaviors reported in competitive scenarios led the public to suspect the view “sports shape great characters.” There are various violations of sports ethics (e.g., insulting, attacking, and cheating) in all levels of sports competition. Moreover, no punishment for those violations not only undermine the value of competitive fairness ethically and morally, but also seriously damage the physical and mental health of athletes. Therefore, in the field of sports psychology, more and more attention has been given to the research on sports ethics and their influencing factors.

Previous findings indicated that psychological factors were considered as the significant roles in affecting athletes’ sports ethics, especially for perfectionism. Perfectionism is usually defined as a personality pursuing perfection and setting the most stringent specification, accompanied by a tendency to overevaluate their own behavior (Hamachek, 1978; Hewitt et al., 1989). Generally, there are two basic dimensions: perfectionistic strivings and perfectionistic concerns. The former one was a positive characteristic that individuals prefer to set themselves higher performance standards and strive for it; by contrast, the later one was a negative characteristic that individuals fear to face devastated evaluation when they do not meet standards (Stoeber and Otto, 2006). It is crucial to distinguish between perfectionistic strivings and perfectionistic concerns when exploring the influencing factors and consequences (Hamachek, 1978), since those two dimensions were totally different and even led to opposing outcomes. Perfectionistic concerns are often positively related to negative processes and outcomes, whereas perfectionistic strivings are often positively related to positive processes and outcomes (or negatively related to negative processes and outcomes) (Stoeber, 2011; Gotwals et al., 2012). A research by Grugan et al. (2020) found that self-directed perfectionism was negatively related to antisocial behavior, whereas other-directed perfectionism was positively related to antisocial behavior. In Madigan et al. (2016), male adolescent athletes were recruited to examine the relationship between perfectionism and doping attitudes. Their findings indicate that perfectionistic strivings were negatively correlated with attitudes in favoring doping. Further, there was no correlation between perfectionistic concerns and attitudes using doping (Madigan et al., 2016). Zhu et al. (2015) found that individuals who worried about making mistakes would receive pressure from coaching and were negatively correlated with prosocial behaviors but positively correlated with antisocial behaviors.

To date, sports ethics have been discussed from different aspects based on the different theories. Notably, the achievement goal theory served as the most extensive theory on this topic. Achievement goal theory was proposed with a 2 × 2 format. The two criteria are the capability of others or self-reference and the value of goal, and the four types would be mastery approach, performance approach, mastery avoidance, and performance avoidance (Elliot, 1999).

Considering previous studies and the theory background above, there was always an indirect relationship between perfectionism and other outcome variables. Therefore, it could be assumed that the mediating role of achievement goals between athletes’ perfectionism and sports ethics might exist and be worth further exploring. Indeed, findings from the previous study have confirmed that Achievement Goals were related to perfectionism and sports ethics, respectively, in sports scenarios. Corrion et al. (2010) found that performance approach and achievement avoidance could positively predict deceptive acceptability, and mastery approach and mastery avoidance could negatively predict deceptive acceptability. In addition, studies found that perfectionistic strivings and perfectionistic concerns had different relationships with achievement goals in sports scenarios (Stoeber et al., 2009; Barkoukis et al., 2011; Chang et al., 2020). Perfectionistic strivings showed a positive correlation with mastery approach and performance approach goals, and perfectionistic concerns showed a positive correlation with mastery avoidance and performance avoidance. Similarly, perfectionistic strivings might mainly focus on approach, whereas perfectionistic concerns might mainly focus on avoidance.

Perfectionism has the potential for affecting sports ethics through achievement goals. That is to say, achievement goals might play a mediating role in the relationship between perfectionism and sports ethics. However, existing studies did not pay attention to this, and such a research question remains unexplored. To fulfill the gap, the aim of this study was to explore the relationship among athletes’ perfectionism, achievement goals, and sports ethics based on achievement goal theory. Hypotheses were given as follows:

(1) Perfectionistic strivings would positively predict the sports ethics of athletes, whereas perfectionistic concerns would negatively predict the sports ethics of athletes.
(2) Mastery approach and mastery avoidance would positively predict athletes’ sports ethics, and performance approach and performance avoidance would negatively predict athletes’ sports ethics.
(3) Mastery approach and performance approach would partially mediate the relationship between perfectionistic strivings and sports ethics, and mastery avoidance and performance avoidance would partially mediate the relationship between perfectionistic concerns and sports ethics.

## Materials and Methods

### Participants

Cluster sampling was employed, and professional athletes in Shanghai and Jiangsu province were recruited. A total of 262 questionnaires were distributed, and 243 valid questionnaires were retrieved with an effective rate of 92.7%. Specifically, there were 128 men (52.7%) and 115 women (47.3%). Further, the participants included 127 (52.3%) first-level athletes, 95 (39.1%) national-elite athletes, and 21 international-elite athletes (8.6%). Sports events included track and field, swimming, cycling, aerobics, basketball, tennis, rowing, etc. The average age of the subjects was 20.5 ± 4.11 years.

### Measures

#### Multidimensional Perfectionism

Two dimensions of perfectionism, perfectionistic strivings and perfectionistic concerns, were measured by the Sport Multidimensional Perfectionism Scale-2 (Sport-MPS-2) and the Multidimensional Inventory of Perfectionism in Sport (MIPS), respectively.

“Striving for perfection” (5 items) and “negative reactions to imperfection” (5 questions) were the two subscales included in MIPS for measuring perfectionistic strivings (Madigan, 2016). “Personal standards” (7 items) and “concern over mistakes” (8 items) were the two subscales included in Sport-MPS-2 for measuring perfectionistic concerns (Gotwals et al., 2010). A five-point Likert scale was used for scoring, ranging from “1” (completely disagree) to “5” (completely agree). The Cronbach’s α coefficients of the four subscales ranged from 0.82 to 0.88. Confirmatory factor analysis (CFA) showed great validity of the two scales (χ2/df = 3.01, RMSEA = 0.06, NFI = 0.91, IFI = 0.94, CFI = 0.94; χ2/df = 3.41, RMSEA = 0.07, NFI = 0.90, IFI = 0.92, CFI = 0.92).

#### Achievement Goals

Achievement goals were measured by the Achievement Goals Questionnaire for Sport (AGQ-S) with 4 subscales, “mastery approach” (3 items), “mastery avoidance” (3 items), “performance approach” (3 items), and “performance avoidance” (3 items) (Conroy et al., 2003). A five-point Likert scale was used for scoring, ranging from “1” (completely disagree) to “5” (completely agree). The Cronbach’s α coefficient of the scale ranged from 0.74 to 0.83. CFA showed great validity of the two scales (χ2/df = 3.61, RMSEA = 0.07, NFI = 0.90, IFI = 0.91, CFI = 0.91).

#### Sports Ethics

Sports ethics was measured by the Multidimensional Sportsmanship Orientation Scale (MSOS) with 4 subscales, “social conventions” (5 items), “rules and officials” (4 items), “respect for opponents” (4 items), and “instrumental aggression” (4 items) (Sun et al., 2013). A five-point Likert scale was used for scoring, ranging from “1” (completely disagree) to “5” (completely agree). The Cronbach’s α coefficients of the four subscales ranged from 0.72 to 0.83. CFA showed great validity of the two scales (χ2/df = 3.69, RMSEA = 0.07, NFI = 0.91, IFI = 0.92, CFI = 0.92).

### Process and Analysis

Each professional sports team was deemed as a unit to conduct the test, and the informed consent from the sports team leader and athletes was obtained before the test. Subjects were informed that the questionnaire was completed anonymously, strictly confidential, and the content was used for scientific research only. The study was approved by the ethical committee of Bio-X center in Shanghai Jiao Tong University, China (reference no. ML16026) and followed the guidelines of the Declaration of Helsinki.

The participants were required to answer each question carefully and independently based on the instructions. It took about 20 min for them to complete all the questions and the questionnaires were collected on spot later.

SPSS 20.0 (IBM Inc., Chicago, IL, United States) and AMOS 20.0 (IBM Inc., Chicago, IL, United States) were used for data analyses. First, Cronbach’s α coefficient and CFA were employed to evaluate the reliability and structural validity of the scale. Second, Pearson’s correlation coefficient was used to examine the relationship among perfectionism, achievement goals, and sports ethics. Finally, structural equation modeling (SEM) was structured by AMOS to evaluate the mediating effect of achievement goals.

## Results

### Descriptive Statistics and Related Analysis

Mean, standard deviation, and correlation coefficient of each variable are listed in Table 1.

**TABLE 1:** Descriptive statistics and correlation analysis for the study variables.

	M	SD	1	2	3	4	5	6	7
1. Perfectionistic strivings	8.26	1.23	1						
2. Perfectionistic concerns	5.96	1.56	–0.02	1					
3. Mastery-approach	4.28	0.70	0.60**	0.13*	1				
4. Performance-approach	3.45	0.80	0.37**	0.43**	0.23**	1			
5. Mastery-avoidance	3.74	0.76	0.25**	0.36**	0.37**	0.29**	1		
6. Performance-avoidance	2.70	0.76	−0.22**	−0.27**	0.63**	0.26**	0.23**	1	
7. Sports ethics	3.63	0.63	0.55**	−0.51**	0.50**	−0.44**	0.41**	−0.52**	1

* is significant at the 0.05 level; ** is significant at the 0.01 level.

Correlation analysis showed that perfectionistic strivings were positively correlated with sports ethics (*r* = 0.55, *p* < 0.01) significantly; perfectionistic concerns were negatively correlated with sports ethics (*r* = −0.51, *p* < 0.01) significantly; perfectionistic strivings were positively correlated with mastery approach (*r* = 0.60, *p* < 0.01) and performance approach (*r* = 0.37, *p* < 0.01) significantly; perfectionistic concerns were positively related to mastery avoidance (*r* = 0.36, *p* < 0.01) and performance avoidance (*r* = −0.27, *p* < 0.01) significantly; mastery approach (*r* = 0.50, *p* < 0.01) and mastery avoidance (*r* = 0.41, *p* < 0.01) were positively correlated with sports ethics significantly; performance approach (*r* = −0.44, *p* < 0.01) and performance avoidance (*r* = −0.52, *p* < 0.01) were negatively correlated with sports ethics significantly.

One-way analysis of variance (ANOVA) showed that there was no significant difference in sports ethics among athletes in different sports events (*F* = 0.063, *p* > 0.05). The pairwise comparison analysis showed that there was no significant difference in the sports ethics of the subjects (*p* > 0.05).

### Test of the Mediation Effect Model

Structural equation modeling was used to test the mediating effect of achievement goals between athletes’ perfectionism and sports ethics.

According to the mediation effect test procedure that suggested by Wen and Ye (2014), two models were structured sequentially: (a) a direct effect model that did not include mediation variables (achievement goals) and (b) a mediation effect model that included mediation variables. The results showed that the direct effects model was well fitted (χ2/df = 3.54, RMSEA = 0.06, NFI = 0.95, IFI = 0.96, CFI = 0.96). Standardized path coefficients of perfectionistic strivings – sports ethics (β = 0.44, *p* < 0.01) and perfectionistic concerns – sports ethics (β = −0.40, *p* < 0.01) were all significant.

**FIGURE 1:**
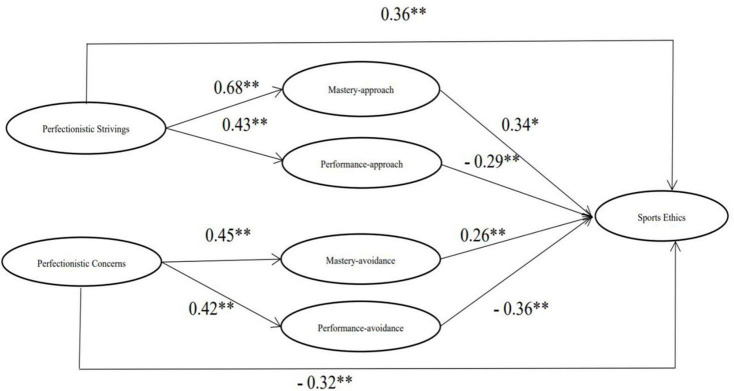
The diagram of the mediation model. * is significant at the 0.05 level; ** is significant at the 0.01 level.

The essential prerequisites for the further test were met. Mastery approach, performance approach, mastery avoidance, and performance avoidance were added as mediation variables to construct a mediating effect model. The mediating effect model was well fitted (χ2/df = 3.94, RMSEA = 0.07, NFI = 0.93, IFI = 0.94, CFI = 0.94). The standardized path coefficients of the mediation effect model have been shown in Figure 1. Direct effects (i.e., perfectionistic strivings – sports ethics – and perfectionistic concerns – sports ethics) were significant in both the direct effect model and the mediation effect model, but the coefficient was lower in the mediation effect model than in the direct effect model, which indicates that there were partial meditations of mastery approach and performance approach on the relationship between perfectionistic strivings and sports ethics, and also of mastery avoidance and performance avoidance on the relationship between perfectionistic concerns and sports ethics.

To further confirm the partial mediation effect of achievement goals in the relationship between perfectionism and sports ethics, the complete mediation effect model was constructed as well. Although the complete mediation effect model was acceptable (χ2/df = 4.16, RMSEA = 0.07, NFI = 0.89, IFI = 0.90, CFI = 0.90), compared with the partial mediation effect model, χ2 increased significantly (Δχ2 = 73.56, Δdf = 2, *p* < 0.01), and the model fit was not the partial one, which showed that the paths, perfectionistic strivings – sports ethics and perfectionistic concerns – sports ethics, were worthwhile, and the partial mediation of achievement goals on the relationship between perfectionism and sports ethics was further confirmed.

## Discussion

The aim of this study was to explore the relationship between multidimensional perfectionism and sports ethics among young athletes, and the mediating role of achievement goals between multidimensional perfectionism and sports ethics might exist.

Findings of this study indicated that perfectionistic strivings could positively predict sports ethics, whereas perfectionistic concerns could negatively predict sports ethics. Stoeber and Otto argued that perfectionistic strivings referred to an inner drive and an adaptive personality setting higher personal standards or being excellent (Stoeber and Otto, 2006). On the contrary, perfectionistic concerns refer to a negative emotional response, worrying about mistakes, fearing failure, and self-criticizing severely, which depends on the performance (Stoeber and Otto, 2006). Different relationship patterns with positive or negative psychological outcomes were attributed to the two different dimensions. Studies showed that perfectionistic strivings were positively correlated with wellness for success, competitive confidence, internal attribution of success, positive emotional responses to great sports performance, and goal orientation (Stoeber et al., 2007, 2008; Chen et al., 2008; Stoeber and Becker, 2008; Sagar and Stoeber, 2009). Nevertheless, perfectionistic concerns were positively associated with failure fear (avoidance of failure), competition anxiety, external attribution of success, goal avoidance, and burnout (Stoeber et al., 2007, 2008; Chen et al., 2008; Stoeber and Becker, 2008; Sagar and Stoeber, 2009). For perfectionistic strivings, perfectionists tried their best to make it with obtaining satisfaction and self-affirmation. Process of being appreciative might be violated if abuse, attack, and deception behaviors exist in the competitive scenarios. But for perfectionistic concerns, perfectionists who overestimated themselves with unrealistic standards might lead to protecting self-worth and avoiding failure and judgment. It might be easier for individuals to generate abuses, attacks, and deception behaviors to shy away from negative outcomes or judgment under standard rules (Gagne, 2003).

Also, the findings indicated that mastery approach and mastery avoidance could positively predict athletes’ sports ethics significantly, and performance -approach and performance avoidance could negatively predict athletes’ sports ethics significantly. In line with the previous studies, the more the athletes focused on mastery goals, the less they agreed with executing unethical behaviors (Barkoukis et al., 2011). The impact of mastery goals on sports ethics did not change with those two dimensions, and the task-focused goals may be a protector for violating behaviors, such as abuse, assault, and deception. It would be easier for athletes pursuing mastery to show compliance with the rules and fair competition, because they care more about task completion and skill improvement than other goals. For them, all rule-violated behaviors would undermine the appreciating process of enhancing skill or mastery of the tasks when they were driven by mastery goals (Corrion et al., 2010). On the other hand, the higher performance goals the athletes held, the more likely they would violate sports ethics, regardless of the use of performance approach or performance avoidance. Nicholls (1989) supported that focusing on achievement goals may lead to a lack of attention to justice, fairness, and the welfare of others.

In addition, the study findings indicated that performance approach and mastery approach could partially mediate the relationship between perfectionistic strivings and sports ethics. In detail, perfectionistic strivings negatively predicted sports ethics through performance approach, but it also positively predicted sports ethics through mastery approach. Consistent with the previous studies, perfectionistic strivings were positively correlated with the orientation of approaching goals (Stoeber et al., 2008). Perfectionistic strivings were an adaptive personality with a tendency setting high standards or an internal drive pursuing excellence (Stoeber and Otto, 2006). Individuals with perfectionistic strivings were usually success-oriented, which meant that they hoped to demonstrate their capability strongly and were more easily to accept performance approach and mastery approach. Sun et al. (2014) indicated that when performance approach got dominant, defeating their opponents was perceived as success and they would make it by anyway, even violating sports ethics. However, when mastery avoidance became dominant, athletes would pay more attention to improvement or mastery of the skills rather than using fraud or aggression behaviors to achieve success. Therefore, when athletes tend to use performance approach, they will be more likely to adopt behaviors violating sports ethics, whereas when athletes tend to use mastery approach, they will have more potential to refuse those unethical behaviors.

Finally, the findings from this study also showed that mastery avoidance and performance avoidance could partially mediate the relationship between perfectionistic concerns and sports ethics. Perfectionistic concerns could negatively predict sports ethics *via* performance avoidance and positively predict sports ethics by mastery avoidance. Previous studies have shown that perfectionistic concerns were positively correlated with mastery avoidance (Stoeber et al., 2008). Perfectionistic concerns were a non-adaptive form of perfectionism, which demonstrated constant worries about mistakes, fears of failure, severe self-criticism, and negative emotional responses (Stoeber and Otto, 2006). Perfectionistic concerns were guided by avoiding failure, trying their best to avoid showing normative incompetence and avoiding introspective incompetence, and individuals are more likely to accept performance avoidance and mastery avoidance. Capability did not change the influence of achievement goals on the acceptability of unethical behaviors (Corrion et al., 2010). When athletes oriented by performance avoidance, they would be more likely to approve the use of unethical behaviors, but when athletes oriented by mastery avoidance goals, they would be more likely to follow the standard rules.

There were some limitations that should be noted. First, the sample size of this study is relatively small. Future studies with larger sample size are needed to avoid bias representation and increase the generalizability of findings. Second, the nature of observational study does not allow the demonstration of causality. Future experimental studies are needed to further examine relationships between those variables.

Since perfectionism and goal orientation execute a greater impact on sports ethics, this study findings may provide guidance on education practice. To prevent athletes from using unethical behaviors, strategies should be developed to reduce perfectionistic concerns and increase their perfectionistic strivings, which could reduce the sense of intimidation and pressure to a certain extent, so that they can continue to enjoy themselves in sports and realize their potential. In addition, mastery approach may be a protective factor for athletes to avoid unethical behaviors. Therefore, coaches and parents should be encouraged to create a mastery environment, such as designing various tasks of difficulty levels, allowing athletes to practice within their own capability, or encouraging them to set specific short-term goals based on their own practice. Also, timely evaluation and recognition of athletes’ progress and achievements will be a great means for cultivating mastery approach and help them succeed.

## Conclusion

Perfectionistic strivings could positively predict sports ethics, whereas perfectionistic concerns could negatively predict sports ethics. Mastery approach and mastery avoidance could positively predict sports ethics, whereas the performance approach and performance avoidance could negatively predict sports ethics. Achievement goal could partially mediate the relationship between perfectionism and sports ethics. Perfectionistic strivings could negatively predict sports ethics through performance approach or positively predict sports ethics through mastery approach. Perfectionistic concerns could negatively predict sports ethics through performance avoidance or could positively predict sports ethics by mastery avoidance.

## Data Availability Statement

The raw data supporting the conclusions of this article will be made available by the authors, without undue reservation.

## Ethics Statement

The studies involving human participants were reviewed and approved by the Bio-X center in Shanghai Jiao Tong University, China. Reference No. ML16026. The patients/participants provided their written informed consent to participate in this study.

## Author Contributions

KS contributed to the conception of the study and performed the manuscript modification. TJ and KS performed the experiment. TJ performed the data analyses and wrote the manuscript. All authors contributed to the article and approved the submitted version.

## Conflict of Interest

The authors declare that the research was conducted in the absence of any commercial or financial relationships that could be construed as a potential conflict of interest.

## Publisher’s Note

All claims expressed in this article are solely those of the authors and do not necessarily represent those of their affiliated organizations, or those of the publisher, the editors and the reviewers. Any product that may be evaluated in this article, or claim that may be made by its manufacturer, is not guaranteed or endorsed by the publisher.
